# Breast-Conserving Surgery in Triple-Negative Breast Cancer: A Retrospective Cohort Study

**DOI:** 10.1155/2023/5431563

**Published:** 2023-01-17

**Authors:** Hengle Zhang, Zunyi Wang, Wei Liu, Peng Wang, Xiaoyu Zhang

**Affiliations:** ^1^Graduate School of Hebei Medical University, Cangzhou Central Hospital Affiliated to Hebei Medical University, Shijiazhuang, China; ^2^Department of Thyroid and Breast Surgery, Cangzhou Central Hospital, Cangzhou, China

## Abstract

**Objectives:**

The aim of the study is to evaluate the efficacy and prognosis of neoadjuvant chemotherapy (NAC) combined with breast-conserving surgery (BCS) in treating triple-negative breast cancer (TNBC) and analyze the influencing factors and predictors of the efficiency and prognosis of NAC.

**Methods:**

A retrospective cohort study was conducted by dividing patients into two groups according to two different therapy methods. With BCS as the exposure factor, 46 cases were assigned to the exposed group and 80 cases to the nonexposed group. We compare the difference in operation-related indicators, postoperative complications, local recurrence rate, distant metastasis rate, and overall survival (OS) rate between the two groups. The factors affecting the efficiency and prognosis of NAC were analyzed by binary logistic regression, and the optimal cutoff value was determined by the area under the ROC curve (AUC). The survival curve was plotted, and the univariate log-rank test was performed to analyze the difference in OS between the two groups. The influencing factors of OS were analyzed by the Cox risk regression model.

**Results:**

NAC + BCS resulted in significantly less intraoperative blood loss, lower incidence of postoperative complications, and shorter operative time and length of hospital stay than that in NAC (*P* < 0.05). There was no significant difference in local recurrence, distant metastasis, or OS between the two groups (*P* > 0.05). Multivariate analysis showed that the clinical stage I and Ki-67 high expression were independent protective factors of the efficacy of NAC. The high expression of Ki-67 and nondecline expression of Ki-67 were independent risk factors of prognosis. Ki-67 high expression was an independent risk factor of OS (*P* < 0.05). The ROC curve showed that the AUC of Ki-67 for NAC efficacy, prognosis, and OS were 0.706, 0.820, and 0.687, respectively, with optimal cutoff values of 25.5%, 29.0%, and 32.5%, respectively. Survival analysis showed that the OS of patients with NAC + BCS was 73.9% and NAC + MRM was 70.0% (*P* > 0.05). In the low expression subgroup of Ki-67, the OS of the two groups were 100.0% and 77.8%, respectively (*P*=0.060). In the high expression subgroup of Ki-67, the OS of the two groups were 53.8% and 63.6%, respectively (*P*=0.419).

**Conclusions:**

NAC + BCS is a good method for treating TNBC, which has an obvious short-term effect and a good long-term prognosis. Clinical stage I and the high expression of Ki-67 are independent protective factors for the efficacy of NAC. The high expression of Ki-67 and nondecline expression of Ki-67 are independent risk factors of prognosis. Ki-67 is a potential predictor for the efficacy, prognosis, and OS of NAC in TNBC patients. The high expression of Ki-67 indicates better NAC efficacy, a poorer prognosis, and a lower OS.

## 1. Introduction

Breast cancer is a high-incidence tumor that seriously threatens the health of women [1–3]. Breast cancer patients with negative results in the tests of ER, PR, and HER-2 are diagnosed with triple-negative breast cancer (TNBC), which has a poor prognosis [4–6]. Although TNBC has no response to conventional treatment, it is sensitive to neoadjuvant chemotherapy (NAC) and significantly improves the clinical benefit rate [7, 8]. At present, modified radical mastectomy (MRM) is the routine operation for operable breast cancer, but research has suggested that breast-conserving surgery (BCS) is considered the preferred strategy for early breast cancer [9]. At the present stage, the treatment of TNBC has aroused great clinical attention, and the emphasis lies on the effective improvement of treatment efficiency. Evidence has revealed significant benefits in short-term efficacy and long-term prognosis for TNBC patients treated with NAC + BCS [10]. To this end, the present study was performed to analyze the efficacy and prognosis of NAC + BCS in treating TNBC and explore the influencing factors and prediction indicators about the efficiency and prognosis of NAC.

## 2. Materials and Methods

### 2.1. Research Objective

A retrospective cohort analysis method was used to collect general clinical data of TNBC patients treated with NAC + MRM or BCS in Cangzhou Central Hospital from January, 2016, to December, 2018. All patients received biopsy to obtain the pathological tissue before treatment, and their immunohistochemical results met the diagnostic criteria for TNBC. A total of 126 patients were collected and assigned to receive either NAC + BCS (exposure group, *n* = 46) or NAC + MRM (nonexposure group, *n* = 80) by using a retrospective cohort study method with BCS as the exposure factor. The patient characteristics between the two groups were comparable (*P* > 0.05) (Table 1).

The inclusion criteria were as follows: (1) aged 26–72; (2) patients without receiving other treatments; (3) the patient was pathologically diagnosed with breast cancer; (4) clinical stage I or II breast cancer; and (5) the diameter of single breast tumor less than 4 cm. According to the inclusion criteria, 140 cases met the inclusion criteria.

The exclusion criteria were as follows: (1) patients with poor compliance; (2) patients who were in pregnancy or lactation; (3) patients who did not meet the diagnostic criteria of TNBC; (4) patients with poor heart, liver, and kidney function who cannot tolerate chemotherapy; (5) patients who had serious adverse reactions and change chemotherapy plan or refuse chemotherapy; (6) patients who found distant metastases before treatment; and (7) patients who had other malignant tumors. Finally, 126 patients were included.

### 2.2. Treatment Methods

In this study, patients were divided into the exposed group and the nonexposed group. Patients in the exposed group received NAC + BCS, and patients in the nonexposed group received NAC + MRM. Neoadjuvant therapy: On day 1, all patients received 30 mg/m^2^ of doxorubicin liposomal (A) and 80 mg/m^2^ of docetaxel (T), referred to as the AT method, 21 days is one cycle, and there are 6 cycles in total. The effect of NAC was evaluated every 2 cycles. Routine symptomatic treatment was performed during NAC. The original chemotherapy was carried out for 4 cycles after the operation.

### 2.3. Immunohistochemistry

Before and after treatment, the expression of Ki-67 was determined by immunohistochemistry (IHC), and the staining ratio of cancer cells (positive cells) was calculated. A proportion of positive cells ≥30% indicated Ki-67 had high expression and <30% indicated Ki-67 had low expression. After NAC, the Ki-67 was divided into a nondecline group (no change and increase) and decline group according to the Ki-67 expression levels.

### 2.4. Observation Indicator

The related indicators between the two groups were compared: (1) surgery-related indicators (intraoperative blood loss, operation time, and length of hospital stay); (2) postoperative complications (tissue edema, skin infection, and postoperative bleeding); and (3) local recurrence rate, distant metastasis rate, and overall survival rate (OS).

### 2.5. Efficacy Criteria and Evaluation

According to the Response Evaluation Criteria for Solid Tumors Version 1.1 [11], complete response (CR): all target lesions disappeared and the diameter of lymph nodes was less than 10 mm; partial response (PR): reduction ratio of the sum of target lesions' diameters exceeds 30%; disease progression (PD): at least 20% increase or the appearance of one or more new lesions; stable disease (SD): the proportion of target lesion reduction or increase did not meet the requirements of PR and PD.

Overall survival rate (OS) was the clinical endpoint, and recurrence and metastasis were the secondary endpoints. The curative effect was divided into the effective group (CR + PR) and the ineffective group (SD + PD). The prognosis was assessed by local recurrence and distant metastasis, and the patients were divided into a good prognosis group and a poor prognosis group.

### 2.6. Follow-Up

Postoperative follow-up was performed by outpatient clinics and telephone calls and terminated in December, 2021. The follow-up was carried out once every 3 months in the first year and once every six months after one year. Overall survival was measured from the day of cancer diagnosis to death or the cutoff date. The overall survival rate referred to the proportion of the total number of survivors by the cutoff date.

### 2.7. Statistical Methods

Data were statistically processed by using SPSS 25.0. The measurement data were expressed as mean ± standard deviation and analyzed using the independent samples *t*-test. The count data were expressed as rate and analyzed using the chi-square test and continuity correction method. A binary logistic regression analysis was used to analyze the independent influencing factors about the efficacy, prognosis, and OS of NAC. The area under the ROC curve (AUC) was used to evaluate the predictive value of Ki-67 for the efficacy, prognosis, and OS of NAC and to determine the optimal cut-off value. The survival curve was plotted, and the difference in OS between groups was analyzed by a univariate log-rank test. The Cox risk regression model was used to analyze the influencing factors of OS. *P* < 0.05 indicated that the difference was statistically significant.

## 3. Results

### 3.1. General Clinicopathological Features

The patients were aged 26–72 years, with 41 cases in <36 years, 57 cases in 36–60 years, and 28 cases >60 years. There were 38 cases in clinical stage I and 88 cases in stage II. There were 34 cases in histological grade I, 68 cases in grade II, and 24 cases in grade III. 55 cases had a tumor diameter that was less than or equal to 2 cm and 71 cases had a tumor diameter that was more than 2 cm. Ki-67 showed low expression in 56 cases and high expression in 70 cases.

### 3.2. Surgery-Related Indicators in the Two Groups

NAC + BCS resulted in significantly less intraoperative blood loss, a lower incidence of postoperative complications, and shorter operative time and length of hospital stay without BCS, and the difference was significant (*P* < 0.05) (Table 2).

### 3.3. Postoperative Complications in the Two Groups

The comparison of postoperative complications in the two groups and the difference was significant (*P* < 0.05) (Table 3).

### 3.4. Long-Term Prognosis of the Two Groups

There was no statistically significant difference between the two groups in local recurrence rate, distant metastasis rate, and overall survival rate (*P* > 0.05) (Table 4).

### 3.5. Analysis of Influencing Factors of NAC Efficacy

Univariate analysis showed that the clinical stage and Ki-67 were both influencing factors of NAC efficacy (*P* < 0.05). Multivariate analysis showed that clinical stage I and high expression of Ki-67 were independent protective factors of NAC efficacy, and the difference was significant (*P* < 0.05) (Tables 5 and 6).

### 3.6. Analysis of Influencing Factors of Prognosis and Overall Survival Rate

Univariate analysis showed that clinical stage, tumor size, Ki-67, and Ki-67 alterations were influencing factors of prognosis, and tumor size, Ki-67, and Ki-67 changes were influencing factors of OS (*P* < 0.05). Multivariate analysis showed that the high expression of Ki-67 and nondecline expression of Ki-67 were independent risk factors of prognosis. The Cox risk regression model showed that only high expression of Ki-67 was an independent risk factor for OS, and the difference was significant (*P* < 0.05) (Tables 7–9).

### 3.7. Determination of the Optimal Cutoff Value of Ki-67

#### 3.7.1. Predictive Value of Ki-67 on the Efficacy of NAC

**Figure 1:**
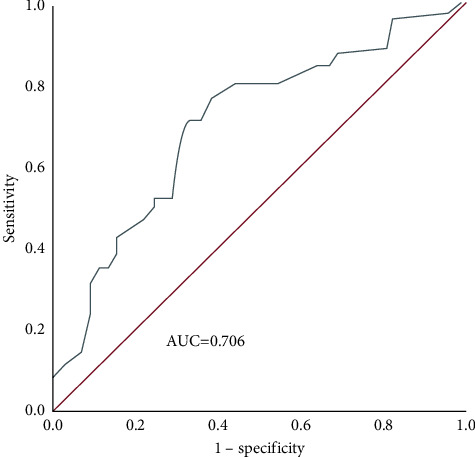
ROC curve of Ki-67 to evaluate the efficacy of NAC.

The ROC curve showed that the AUC of Ki-67 for evaluating the efficacy of NAC was 0.706 (95% CI: 0.612–0.801), the optimal cutoff value was 25.5%, the sensitivity was 76.5%, and the specificity was 62.2% (Figure 1).

#### 3.7.2. Predictive Value of Ki-67 on Prognosis

**Figure 2:**
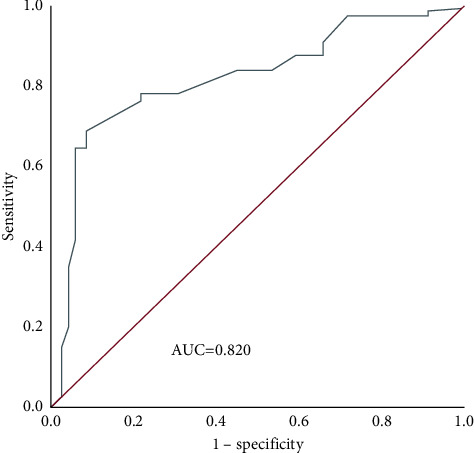
ROC curve of Ki-67 in evaluating the prognosis.

The AUC of Ki-67 for evaluating the prognosis was 0.820 (95% CI: 0.744–0.897), the optimal cutoff value was 29.0%, the sensitivity was 68.4%, and the specificity was 92.0% (Figure 2).

#### 3.7.3. Predictive Value of Ki-67 on OS

**Figure 3:**
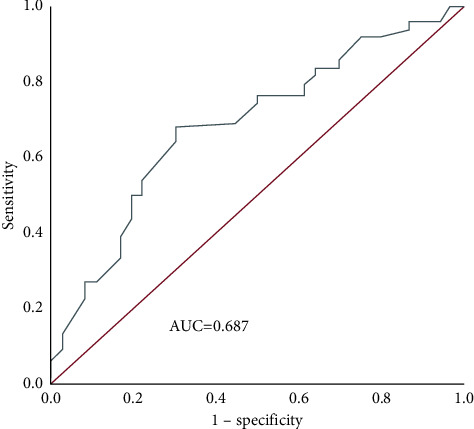
ROC curve of Ki-67 to evaluate OS.

The AUC of Ki-67 for evaluating OS in TNBC patients was 0.687 (95% CI: 0.586–0.788), the optimal cutoff value was 32.5%, the sensitivity was 67.8%, and the specificity was 69.4% (Figure 3).

### 3.8. Survival Analysis

**Figure 4:**
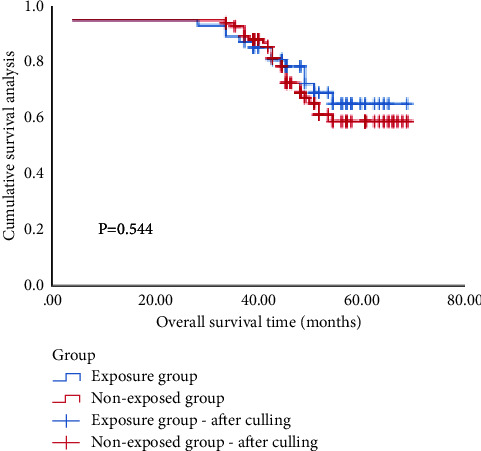
Overall survival curve of two groups of patients.

**Figure 5:**
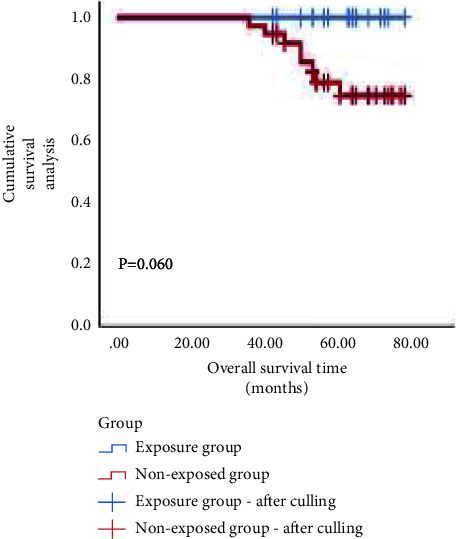
Overall survival curve of two groups of patients with low Ki-67 expression.

**Figure 6:**
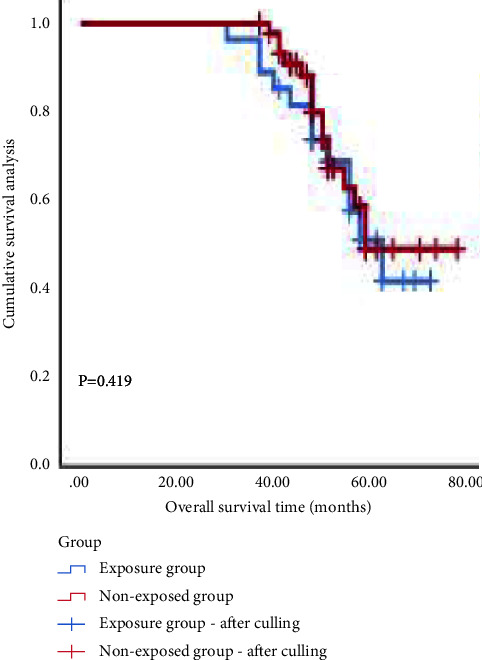
Overall survival curve of two groups of patients with high Ki-67 expression.

Survival analysis showed that as of December, 2021, the median follow-up time was 49 months; there were 23 cases of local recurrence, 27 cases of distant metastasis, and 36 cases of death. The overall survival time was 27–72 months, and the overall survival rate was 71.4%. The OS of the two groups was 73.9% and 70.0%, respectively (*χ*^2^ = 0.369, *P* > 0.05). As Ki-67 was an independent influencing factor of OS, a single-factor hierarchical analysis was performed. In the low expression subgroup of Ki-67, the OS of the two groups were 100.0% and 77.8% (*χ*^2^ = 3.529, *P*=0.060). In the high expression subgroup of Ki-67, the OS of the two groups were 53.8% and 63.6% (*χ*^2^ = 0.653, *P*=0.419). (Figures 4–6).

## 4. Discussion

TNBC, which accounts for 10%–20% of breast cancer, is insensitive to conventional local treatment and has a poor prognosis [12]. Research has shown that NAC based on anthracyclines combined with taxanes has a favorable overall response and demonstrates good potential as an effective treatment strategy for breast cancer [13]. NAC can effectively improve the therapeutic effect of TNBC [7, 8]. Breast-conserving surgery is the preferred scheme for early breast cancer [9]. The author will discuss the following 5 aspects and summarize the full text.

### 4.1. Analysis of Short-Term Efficacy and Long-Term Prognosis in the Two Groups

Our study found that the exposed group was superior to the nonexposed group in terms of surgical indicators and postoperative complications, indicating that NAC can provide a good material basis for surgery, reduce the risk of postoperative complications, and improve the efficacy of BCS. Our study found that the long-term prognosis of the exposed group was slightly better than that of the exposed group (*P* > 0.05), but TNBC patients who had received the BCS regimen achieved a higher clinical benefit rate. For operable patients, NAC + BCS is safe and feasible [14]. Therefore, NAC + BCS can effectively improve the clinical benefit rate of TNBC patients.

### 4.2. Analysis of Influencing Factors of NAC Efficacy, Prognosis, and OS

Our study found that clinical stage I and high expression of Ki-67 were independent protective factors for the efficacy of NAC (*P* < 0.05). Studies have found that the early clinical stage of breast cancer and high expression of Ki-67 contribute to better NAC efficacy [15–17], which is consistent with the results of our study. Our study found that the high expression of Ki-67 and nondecline expression of Ki-67 were both independent risk factors for the prognosis of TNBC. Studies have revealed that high expression of Ki67 in residual lesions of locally advanced breast cancer after surgery was associated with poor recurrence-free survival (*P*=0.004) and decreased Ki67 expression (the degree of reduction is greater than 12.5%) after NAC was associated with longer recurrence-free survival (*P*=0.007) [18]. Our study also identified high expression of Ki-67 as an independent risk factor for OS. It has been reported that high expression of Ki-67 in TNBC patients is associated with low OS [19]. Thus, our study concluded that Ki-67 expression was related to the efficacy, prognosis, and OS of NAC, and TNBC patients with high expression of Ki-67 were predisposed to good NAC efficacy but poor long-term prognosis.

### 4.3. The Predictive Value of Ki-67 for NAC Efficacy, Prognosis, and OS

Our study found that Ki-67 had a certain predictive potential for the efficacy, prognosis, and OS of NAC, and the high expression of Ki-67 might suggest better efficacy, a worse prognosis, and a lower OS of NAC. It has been shown that the high expression of Ki-67 could predict a poor long-term prognosis [19] and better efficacy of NAC [20], which is consistent with the results of our study. Therefore, we believe that Ki-67 may act as a predictor of efficacy and long-term prognosis of NAC in TNBC patients.

### 4.4. Survival Analysis of Ki-67 Expression Subgroup

Our study found that the difference in OS between the two groups was not statistically significant, but Ki-67 was an independent influencing factor of OS. Univariate stratified analysis found that in the low expression subgroup of Ki-67, the OS of the two groups were 100.0% and 77.8%, respectively, and in the high expression subgroup of Ki-67, the OS of the two groups were 53.8% and 63.6%, respectively. Therefore, our study considers that TNBC patients with a low expression subgroup of Ki-67 have a higher OS and may benefit from breast-conserving surgery; however, TNBC patients with a high expression subgroup of Ki-67 have a lower OS, and they are not suitable for breast-conserving surgery.

### 4.5. Limitation

Compared with prospective cohort studies, the limitations of the current study include the small sample size, short follow-up, and data bias. Therefore, more rigorous prospective studies will be carried out to further verify the results of our study.

## 5. Conclusions

NAC combined with BCS is a promising regimen for the treatment of TNBC, providing favorable short-term therapeutic benefits, an enhanced prognosis, and an improved OS. Clinical stage I and the high expression of Ki-67 are independent protective factors for the efficacy of NAC. The high expression of Ki-67 and nondecline expression of Ki-67 are independent risk factors of prognosis. Ki-67 is a potential predictor of NAC efficacy, prognosis, and OS in TNBC patients. The high expression of Ki-67 indicates better NAC efficacy, a poorer prognosis, and a lower OS.

## 6. Consent

All patients signed an informed consent.

## Figures and Tables

**Table 1:** Comparison of general data of two groups of patients.

Group	Number	Age (years, *x* ± *s*)	*Tumor size (n, %)*		*Clinical stage (n, %)*		*Ki-67 (n, %)*		*Histological grade (n, %)*		
			≤2 cm	>2 cm	Stage I	Stage II	<30%	≥30%	Grade I	Grade II	Grade III
Exposure group	46	44.72 ± 13.75	24 (52.2)	22 (47.8)	16 (34.8)	30 (65.2)	20 (43.5)	26 (56.5)	15(32.6)	21 (45.7)	10 (21.7)
Nonexposure group	80	47.24 ± 12.45	31(38.8)	49 (61.3)	22(27.5)	58 (72.5)	36 (45.0)	44 (55.0)	19 (23.8)	47 (58.8)	14 (17.5)
*t*/*χ*^2^		−1.053	2.140		0.735		0.027		2.053		
*P*		0.295	0.144		0.391		0.869		0.358		

**Table 2:** Surgery-related indicators in the two groups (*x* ± *s*).

Group	Number of cases	Intraoperative blood loss (ml)	Operation time (min)	Hospitalization time (d)
Exposure group	46	51.37 ± 7.15	49.78 ± 7.55	8.00 ± 1.17
Nonexposure group	80	101.74 ± 8.64	87.30 ± 9.71	17.14 ± 2.40
*T*		−33.47	−22.56	−24.22
*P*		0.001	0.001	0.001

**Table 3:** Postoperative complications in the two groups (*n*, %).

Group	Number of cases	Tissue edema	Postoperative bleeding	Skin infection	Total
Exposure group	46	2 (4.3)	2 (4.3)	1 (2.2)	5 (10.9)
Nonexposure group	80	12 (15.0)	10 (12.5)	7 (8.8)	29 (36.3)
*χ * ^2^					9.549
*P*					0.002

**Table 4:** The long-term prognosis of the two groups (*n*, %).

Group	Number of cases	Local recurrence rate	Distant metastasis rate	Overall survival rate
Exposure group	46	8 (17.4)	9 (19.6)	34 (73.9)
Nonexposure group	80	15 (18.8)	18 (22.5)	56 (70.0)
*χ * ^2^		0.036	0.149	0.219
*P*		0.849	0.699	0.640

**Table 5:** Univariate analysis of NAC efficacy (*n*, %).

Variable	Number	Effective	*χ * ^2^	*P*
*Ki-67*			16.94	0.001
<30%	56	25 (44.6)		
≥30%	70	56 (80.0)		

*Clinical stage*			12.06	0.001
Stage I	38	33 (86.8)		
Stage II	88	48 (54.5)		

*Tumor size*			1.584	0.208
≤2 cm	55	32 (58.2)		
>2 cm	71	49 (69.0)		

*Age*			0.201	0.905
≤35 years	41	26 (63.4)		
35–60 years	57	36 (63.2)		
>60 years	28	19 (67.9)		

*Histological grade*			4.931	0.085
Grade I	34	25 (73.5)		
Grade II	68	45 (66.2)		
Grade III	24	11 (45.8)		

**Table 6:** Multivariate analysis of NAC efficacy.

Variable	Β	*s *_*x*_	Wald	OR	95% CI	*P*
Clinical stage	−2.190	0.583	14.10	0.112	0.036–0.351	0.001
Ki-67	1.999	0.456	19.19	7.383	3.018–18.06	0.001

**Table 7:** Univariate analysis of prognosis and overall survival rate (*n*, %).

Variable	Number	Good prognosis	*χ* ^2^	*P*	Number	OS	*χ* ^2^	*P*
*Ki-67*			44.59	0.001			14.65	0.001
<30%	56	52 (92.9)			56	48 (85.7)		
≥30%	70	24 (34.3)			70	42 (60.0)		

*Clinical stage*			4.061	0.044			3.447	0.063
Stage I	38	28 (73.7)			38	32 (84.2)		
Stage II	88	48 (54.5)			88	58 (65.9)		

*Tumor size*			10.50	0.001			11.32	0.001
≤2 cm	55	42 (76.4)			55	47 (85.5)		
>2 cm	71	34 (47.9)			71	43 (60.6)		

*Age*			0.517	0.772			1.489	0.475
≤35 years	41	23 (56.1)			41	30 (73.2)		
35–60 years	57	35 (61.4)			57	38 (66.7)		
>60 years	28	18 (64.3)			28	22 (78.6)		

*Histological grade*			0.561	0.755			0.344	0.842
Grade I	34	19 (55.9)			34	23 (67.6)		
Grade II	68	43 (63.2)			68	50 (73.5)		
Grade III	24	14 (58.3)			24	17 (78.6)		

*Curative effect*			0.003	0.957			0.075	0.784
Invalid	45	27 (60.0)			45	31 (68.9)		
Valid	81	49 (60.5)			81	59 (72.8)		

*Ki-67 variation*			16.52	0.001			4.847	0.028
Nondecline	46	17 (37.0)			46	28 (60.9)		
Decline	80	59 (73.8)			80	62 (77.5)		

**Table 8:** Multivariate analysis of prognosis.

Variable	*β*	*S*_*x*_	Wald	OR	95% CI	*P*
Clinical stage	−0.802	0.619	1.677	0.448	0.133–1.510	0.195
Ki-67	−2.932	0.603	23.64	0.053	0.016–0.174	0.001
Tumor size	−4.636	0.598	0.599	0.630	0.195–2.032	0.439
Ki-67 variation	1.375	0.504	7.447	3.954	1.473–10.62	0.006

**Table 9:** Multivariate analysis of the overall survival rate.

Variable	*β*	*s*_*x*_	Wald	HR	95% CI	*P*
Tumor size	0.704	0.459	2.347	2.021	0.822–4.972	0.126
Ki-67	1.001	0.443	5.111	2.720	1.142–6.476	0.024
Ki-67 variation	−0.422	0.347	1.482	0.656	0.332–1.294	0.223
Stage	0.406	0.484	0.701	1.500	0.581–3.878	0.402

## Data Availability

The datasets of this study are available from the first author upon reasonable request.
